# Effects of Periplocoside P from *Periploca sepium* on the Midgut Transmembrane Potential of *Mythimna separata* Larvae

**DOI:** 10.1038/srep36982

**Published:** 2016-11-11

**Authors:** YingYing Wang, Zhijun Qi, Meng Qi, Zhaonong Hu, Wenjun Wu

**Affiliations:** 1 Institute of Pesticide Science, Northwest A&F University, Yangling, Shaanxi 712100, China; 2 Key Laboratory of Botanical Pesticide R&D in Shaanxi Province, Shaanxi 712100, China

## Abstract

Periplocoside P (PSP) isolated from the root bark of *Periploca sepium* contains a pregnane glycoside skeleton and possesses high insecticidal properties. Preliminary studies indicated that PSP disrupts epithelial functions in the midgut of lepidopteran larvae. In the present study, we examined the effects of PSP on the apical and basolateral membrane voltages, *V*_a_ and *V*_bl_, respectively, of cells from (1) midguts isolated from the larvae of the oriental armyworm *Mythimna separata* that were *in vitro* incubated with toxins and (2) midguts isolated from *M. separata* larvae force-fed with PSP. We compared the effects of PSP with the effects of the *Bacillus thuringiensis* toxin Cry1Ab and inactive periplocoside E (PSE) on the midgut epithelial cells. The results showed that *V*_a_ rapidly decreased in the presence of PSP in a time- and dose-dependent manner, similar to the effects of Cry1Ab. By contrast, PSE did not affect the *V*_a_ and *V*_bl_. Additionally, PSP did not influence the *V*_bl_. Given these results, we speculate that PSP may modulate transport mechanisms at the apical membrane of the midgut epithelial cells by inhibiting the V-type H^+^ ATPase.

Plant-derived natural products are the source of most active ingredients in insecticides, such as physostigmine^1^, pyrethrin^2^,^3^, nicotine^4^, rotenone^5^, and ryanodine^6^. These natural products often exhibit different modes of action, which is particularly important because pests continue to evolve in their resistance to current insecticides^7^. Thus, plant-derived natural products still have a high potential in the discovery and development of new pesticides^8^,^9^.

*Periploca sepium* Bunge, the deciduous shrub of the family Asclepiadaceae, is a traditional medicinal herb used for the treatment of autoimmune diseases, particularly rheumatoid arthritis, in China^10^,^11^. Extracts of the root bark of the shrub possess insecticidal properties and are used to control several lepidopteran insect pests, such as the diamondback moth *Plutella xylostella* and the cabbage caterpillar *Pieris rapae* in China^12^,^13^. In addition, Li *et al*.^14^ reported that three compounds from *P. sepium* display insecticidal activity against the red imported fire ant *Solenopsis invicta*. The oil of the *P. sepium* root bark has been reported to cause strong contact toxicity to the fruit fly *Drosophila melanogaster*^11^. The above-mentioned reports suggest that *P. sepium* is a rich source of insecticides. In particular, new periplocoside compounds with pregnane glycoside skeletons have been isolated from the root bark of *P. sepium*, such as periplocoside A, periplocoside P (PSP), periplocoside T and periplocoside NW (PSNW), with reported insecticidal activities^13^,^14^,^15^,^16^,^17^,^18^. PSP exhibits a high stomach toxicity against the third larval stage of the oriental army worm *Mythimna separata*, with an LC_50_ value of 110 mg·L^−1^ at 48 h^18^. Preliminary histopathological and ultrastructural studies showed that periplocosides disrupt the midgut epithelial cells of lepidopteran larvae^19^,^20^. According to the results from fluorescence localization and immunolocalization studies of PSNW, the main mechanism of toxicity is by damage to the midgut cell and organellar membranes in *M. separata* larvae; the deduced binding site is the cell membrane of the midgut epithelial cells^20^,^21^. Alternatively, the results of protease activity-determination demonstrated that PSP can activate the midgut proteases of *M. separata*, particularly the trypsin-like protease^22^. However, the mode of action of PSP or other periplocosides remains unknown.

The physiology of the larval midgut epithelium of lepidopteran insects is characterized by the strong secretory transport of K^+^ from the hemolymph to the midgut lumen^23^,^24^. Fundamental to the transepithelial secretion of K^+^ is the vacuolar-type H^+^-ATPase coupled to an electrogenic K^+^/H^+^ antiporter that are both located at the apical membrane of the midgut goblet cells^25^,^26^,^27^,^28^. In the present study, we used standard microelectrodes to measure the membrane potential in epithelial cells of the midguts isolated from the larvae of *M. separata* as previously described^29^,^30^,^31^,^32^. The membrane potential of epithelial cells reflects the transport properties of the epithelium; hence, we measured the effects of PSP on the membrane potential of apical and basolateral cells.

Our results indicated that PSP inhibits the apical membrane voltage of the midgut epithelium of *M. separata* and might be the first direct evidence that the apical membrane of the midgut epithelium is the action site of PSP. These findings revealed potential targets of the actions of periplocosides and laid a foundation for the further elucidation of the molecular mechanism of action of this botanical insecticide.

## Results

### Effects of orally administered PSP on midgut transmembrane potential

**Figure 1:**
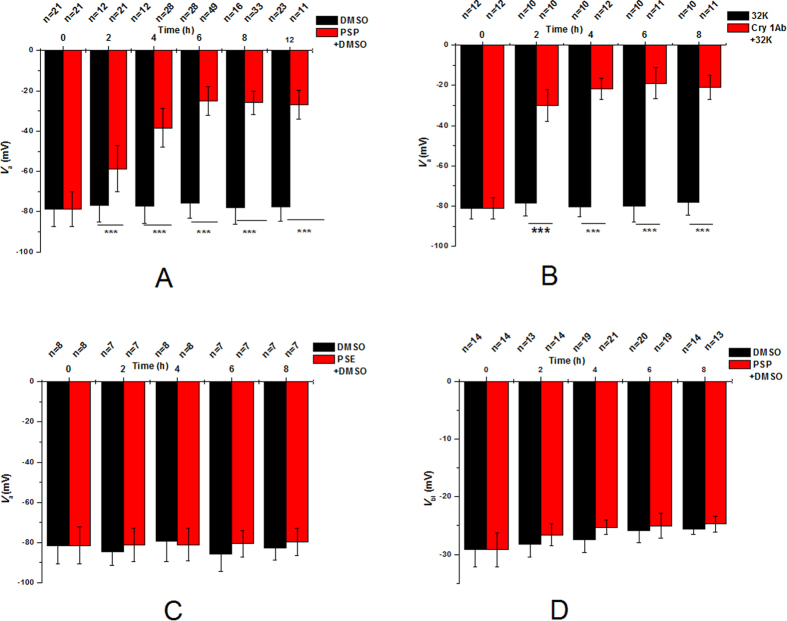
Effects of orally administered PSP on the *V*_a_ and *V*_bl_ of *M. separata* larval midgut. Sixth instar larvae were fed with 1 μL of DMSO containing 20 μg of either test compound or 1 μL of the 32 K solution containing 1 μg activated Cry 1Ab, and the *V*_a_ and *V*_bl_ were measured with time. The control was fed only with 1 μL DMSO or 32 K solution. A-C display the *V*_a_ of PSP, Cry 1Ab, and PSP, respectively; D shows the *V*_bl_ of PSP. *V*_a_ or *V*_bl_ refers to the mean membrane potential measured when the values remain stable over 5 min. The values are expressed as the mean ± SEM for 7 to 28 independent experiments. Three asterisks indicate significant differences by Student’s *t-*test (*P* < 0.001).

The apical membrane potential (*V*_a_) and basolateral membrane potential (*V*_bl_) of *M. separata* midgut epithelial cells were measured after force-feeding the sixth-instar larvae with 1 μL of dimethyl sulfoxide (DMSO) containing 20 μg of either test compound or 1 μL of the 32 K solution containing 1 μg of activated Cry 1Ab for 2 h to 12 h (Fig. 1).

Feeding with 1 μL of only DMSO did not affect the *V*_a_ (−81.3 ± 7.4 mV, *n* = 21) at different periods (Fig. 1A). By contrast, the *V*_a_ (−58.7 ± 11.5 mV, *n* = 21) at 2 h was significantly reduced from the initial value (−76.8 ± 8.3 mV, *n* = 12) after the ingestion of 1 μL of DMSO with PSP. The *V*_a_ increasingly deteriorated toward the positive direction with time during PSP feeding. The *V*_a_ was −38.4 ± 9.6 mV (*n* = 28) after 4 h, −25.1 ± 7.2 mV (*n* = 49) after 6 h, −25.9 ± 5.89 mV (*n* = 33) after 8 h, and −27.1 ± 7.1 mV (*n* = 11) after 12 h.

The Cry1Ab-fed *M. separata* larvae exhibited similar effects on the *V*_a_ of midgut epithelial cells (Fig. 1B). Moreover, the *V*_a_ was −81.1 ± 5.2 mV (*n* = 12) and stable for up to 8 h in the larvae fed with only the 32 K solution (Fig. 1B). However, after the larvae ingested Cry 1Ab, the *V*_a_ decreased rapidly. After 2, 4, 6, and 8 h of force-feeding with 32 K containing Cry1Ab, *V*_a_ decreased to −30.1 ± 7.8 mV (*n* = 10), −21.7 ± 5.4 mV (*n* = 12), −19.0 ± 7.7 mV (*n* = 11), and −21.1 ± 6.2 mV (*n* = 11), respectively (Fig. 1B). By contrast, periplocoside E (PSE), which does not hold any insecticidal activity against the *M. separata* larvae^32^, did not influence the *V*_a_ (Fig. 1C) and displayed trends similar to those in the control group.

The basolateral membrane voltage (*V*_bl_) of the midguts isolated from the larvae under control conditions was −29.2 ± 2.9 mV (*n* = 14) and did not significantly change in the larvae that ingested PSP (Fig. 1D). Similarly, Cry1Ab exerted no effect on *V*_bl_ as previously reported^27^,^31^.

Accordingly, there was no significant change in the transepithelial voltages (*V*_t_) when only solution was added (DMSO or 32 K). The *V*_t_ following the different treatments are shown in Table 1. The *V*_t_ gradually decreased with time and approached zero for both the PSP and Cry1Ab treatments. The inactive PSE did not significantly influence the *V*_t_. This result further illustrates that the insecticidal activity of PSP is correlated to *V*_t_ changes.

### Effects of PSP incubated with midgut cells on transmembrane potential

**Figure 2:**
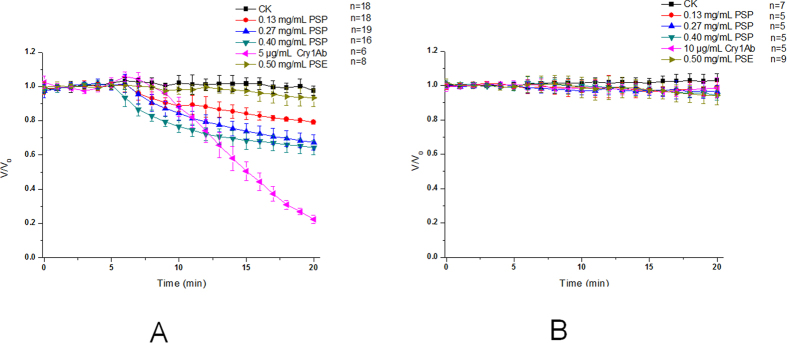
Effects of incubated PSP on the *V*_a_ (**A**) and *V*_bl_ (**B**) of midgut cells from *M. separata* larvae. When the membrane was stable for over 5 min, 0.4 mL of 32 K solution containing the test compounds of interest was directly added to the bath to replace a similar volume solution. The bath concentrations of PSP were 0.0, 0.13, 0.27 or 0.40 mg/mL, and that of PSE was 0.50 mg/mL. Cry1Ab was adopted as the positive control; its concentration was 5.0 μg/mL for *V*_a_ or 10.0 μg/mL for *V*_bl_. *V* refers to *V*_a_ or *V*_b_ measured at the indicated times, and *V*_o_ is the mean membrane potential measured during the 5 min stable condition before the addition of the test compounds. Data are presented as the mean ± SEM for 5 to 18 independent experiments.

Figure 2 shows the effects of the different concentrations of PSP given by bath incubation on the *V*_a_ and *V*_bl_ of the isolated midgut epithelial cells from *M. separata* larvae. Three concentrations of 0.13 mg/mL to 0.4 mg/mL PSP caused a concentration-dependent depolarization of *V*_a_ (Fig. 2A), with the membrane potential depolarized by approximately 15.9 ± 3.4%, 26.3 ± 4.7%, and 31.7 ± 4.9% after 10 min, and 20.9 ± 1.1%, 32.7 ± 4.5%, and 35.8 ± 4.3% after 15 min. By contrast, PSP only slightly influenced the *V*_bl,_ even at 0.4 μg/mL (Fig. 2B).

As the positive control, activated Cry1Ab affected the *V*_a_ and *V*_bl_ to a similar extent as the force-fed PSP. The activated Cry1Ab also depolarized the midgut epithelial cell membrane potential *V*_a_ at a concentration of 5 μg/mL (Fig. 2A). The rate of the depolarization of the apical membrane potential induced by Cry1Ab was higher than that of PSP; the necessary time to reduce membrane potential by 50% was reached after approximately 9.8 ± 0.4 min. However, PSP was faster in response time than Cry1Ab.

Alternatively, the 10 μg/mL concentration of Cry1Ab applied to the basolateral side of the epithelium cells did not influence *V*_bl_ (Fig. 2B). Moreover, 0.5 mg/mL PSE, known to lack insecticidal activity, did not affect either the *V*_a_ or *V*_bl_ (Fig. 2A,B).

## Discussion

In the present study, we examined the effects of PSP on the apical and basolateral membrane potentials. The *V*_a_ and *V*_bl_ of the midgut epithelial cells under both force-feeding and *in vitro* incubation with PSP were obtained and compared with the effects of PSE and the *Bt* toxin Cry1Ab. PSE possesses the same molecular structure as PSP, except for one substituent, and holds no insecticidal activity against lepidopteran larvae^22^. Our findings revealed that PSP inhibits the apical membrane voltage of the midgut epithelial cells of *M. separata*, providing the first direct evidence that the apical membrane of midgut epithelial cells plays a critical role in PSP toxicity.

In general, the transmembrane electrical potential difference is 140 mV or greater and can be measured at the apical side of the columnar cells of lepidopteran larvae^33^. Our results showed that the normal *V*_a_ of the *M. separata* larval midgut cells bathed in the standard 32 K solution was approximately −80 mV and remained stable for up to 30 min. The *V*_a_ from our study was consistent with previous reports of transmembrane voltages in lepidopteran larvae^29^,^30^,^31^,^32^.

The effects of Cry1Ab on the *V*_a_ of *M. separata* larvae midgut epithelial cells were also consistent with the previous results in *Lymantria dispar, Bombyx mori*, and *Manduca sexta* larval midguts^29^,^30^ namely, Cry1Ab significantly depolarized the apical membrane potential. After incubation with 5 μg/mL of Cry1Ab, the *V*_a_ depolarized by approximately 50% after about 10 min. Cry1Ab exerted no significant effect on the basolateral membrane potential. The activated toxin Cry1Ab is well known to bind to specific receptors and form pores in the apical membranes of midgut epithelial cells. These pores abolish ionic gradients and cause the lysis of epithelial cells, leading to insect death^29^,^34^.

Similar to the effect of Cry1Ab, the effect of the depolarization of the apical membrane potential by the PSP of midgut epithelial cells from *M. separata* larvae was concentration- and time-dependent. Notably, PSP was more potent when applied on the apical membrane rather than on the basal membrane of the midgut epithelial cells. This finding suggests that PSP may function by interacting with the apical membrane of the midgut epithelium.

Compared with PSP, PSE is inactive against *M. separata*. We found that PSP significantly affected *V*_a_ under both force-feeding and *in vitro* incubation, but PSE did not influence *V*_a_ even at high concentrations. Consistent with the bioassay result, PSP acted on the apical membranes of the midgut epithelium when the insects were fed with the natural insecticide. PSP influenced the transmembrane ion transport of the insect’s apical membrane, resulting in abnormal transmembrane potentials, which then affected the normal physiological and biochemical functions of the cells. The effects eventually led to ion balance disorder on both sides of the apical membrane, causing midgut cell edema, abdominal swelling, and intestinal rupture until insect death. Because active PSP, rather than inactive PSE, can directly affect the ionic permeability of the apical membrane, membrane potential measurements provide a rapid and sensitive *in vitro* assay for insecticidal potency in their normal target cells.

In accordance with the mode of action of Cry1A toxins, the activated Cry1Ab binds to aminopeptidase N or alkaline phosphatase, which is involved in driving the activated Cry1Ab into the apical membrane where the pre-pore complex is converted into a membrane-inserted pore that finally leads to ion leakage, cell lysis, and insect death^35^,^36^,^37^. PSP is a small molecular compound containing a pregnane glycoside skeleton. Thus, PSP-induced pore formation in the apical membrane, such as that by *Bt* toxin, is theoretically impossible. In general, the transmembrane electrical potential difference and ion/nutrition transport are based on the function of V-type H^+^ ATPase and V-type H^+^ ATPase coupled with the K^+^/H^+^ antiporter in the apical membrane of the midgut epithelium goblet cells of lepidopteran larvae^22^,^25^,^26^,^33^. Our results showed that PSP could directly act on the apical membrane and depolarize the transmembrane potential. A previous study showed that PSP inhibits transepithelial electrolyte and fluid secretion in the Malpighian tubules of the yellow fever mosquito *Aedes aegypti* by inhibiting the V-type H^+^ ATPase^38^. PSP could significantly inhibit the total ATPase activity similar to the inhibition by bafilomycin, which is a known inhibitor of the V-type H^+^ ATPase. Therefore, based on the above analysis and discussion, we can speculate that the most important target of PSP may be the V-type H^+^ ATPase. PSP may potentially disrupt the ionic and chemical homeostasis in midgut epithelial cells and eventually the whole midgut through the cooperative activity of goblet and columnar cells, causing irreversible damage to the midgut.

Alternatively, *Bt* has been reported to potentially act on the V-type H^+^ ATPase and directly block various types of transmembrane transporters^39^. By comparing the effects of Cry1Ab and PSP on the transmembrane potential of the apical membrane of *M. separata*, we found that the effects of PSP incubated with the midgut cell membrane were less efficient than those of Cry1Ab. This finding further illustrates that the effect of Cry1Ab on the membrane potential mainly lies in pore formation in the midgut apical membrane. Such a phenomenon facilitates the ion imbalance across the apical membrane and more easily depolarizes the potential. By contrast, PSP may act on the V-type H^+^ ATPase. Although PSP’s *in vitro* efficiency is less than that of Cry1Ab, the former’s response time in directly acting on the apical membrane potential is faster, and its effects on physiological function are particularly significant. The similarity of the changes in the apical membrane potentials at different times after feeding with PSP and the changes observed in the *Bt* bioassay also illustrate this point.

To our knowledge, PSP is the first small-molecule compound reported as an insecticide that targets the V-type H^+^ ATPase. Even so, we cannot rule out the role of PSP in other important insect targets, such as its previously reported ability to activate trypsin activity^22^. However, additional efforts are needed to elucidate whether PSP involves multiple targets or only acts on only the midgut apical membrane transporter to cause the subsequent effects.

Given our results, we consider that PSP may act on the larval midgut apical membrane of *M. separata*, particularly the V-type H^+^ ATPase of midgut goblet cells, to disrupt the electrochemical gradient and thus abolish the capacity of the cells to transport solutes, which is essential in maintaining the equilibration of the pH between the cytoplasm and the highly alkaline content of the lumen. Such disturbances may lead to the disruption of the midgut epithelium.

## Materials and Methods

### Insects

Laboratory-adapted *M. separata* were provided by the Institute of Pesticide Science, Northwest A&F University (NWAFU), China. The population was reared under laboratory conditions (25 °C, 75% to 80% relative humidity, and a 16 h light: 8 h dark photoperiod) and was not exposed to any pesticides prior to the experiment. The larvae were reared on wheat leaves. The actively feeding sixth-instar larvae were used for electrophysiological experiments.

### Solutions

Experiments were conducted in the standard 32 K solution, which was composed of 32 mM KCl, 5 mM CaCl_2_, 5 mM MgCl_2_, 166 mM sucrose, and 5 mM Tris–HCl (pH 8.0)^23^. The solution was filtered through a membrane with a 0.2-mm pore diameter (Gelman Science, Inc., Ann Arbor, MI, USA).

### Toxins

Trypsin-activated, purified Cry1Ab protein standard was purchased from Envirologix (Portland, ME, USA). Stock solutions of 2 mg/mL Cry1Ab were prepared in 25 mM Tris–HCl (pH 9.4) and stored at 4 °C. The stock solutions were diluted daily to the appropriate concentration in the 32 K solution.

### Test compounds

**Figure 3:**
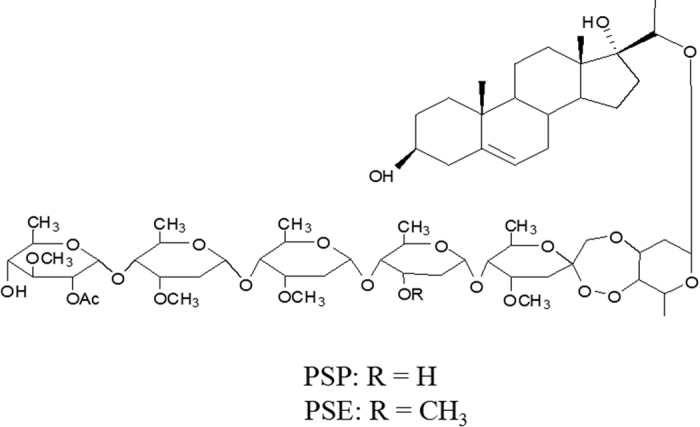
The structure of testing periplocosides.

PSP and PSE (technical grade, >95%) were isolated and provided by the Institute of Pesticide Science,NWAFU. The structure of PSP is pregn-5-ene-3β,17α,20(S)-triol-3-hydroxy-20-O-2-O-acetyl-β-digitalopyranosyl(1 → 4)-O-β-cymaropyranosyl(1 → 4)-O-β-cymaropyranosyl(1 → 4)-O-β-digitopyranosyl(1 → 4)-O-3,7-dideoxy-4-methoxy-2-heptulopyranosyl(2 → 4)-dioxy-(1 → 3)-O-β-canaropyranoside, and both compound structures are displayed in Fig. 3^17^,^40^. Stock solutions of PSP and PSE were prepared at a concentration of 20 mg/mL in DMSO and stored at 4 °C.

### Treatment

To evaluate the effects of PSP on *M. separata* larvae, each sixth-instar larva of *M. separata* was fasted for 8 h and then force-fed with 1 μL of DMSO containing 20 μg of either test compound or 1 μL of the 32 K solution containing 1 μg of activated Cry 1Ab^31^,^41^. DMSO or 32 K solution was administered to the control larvae. The treated *M. separata* larvae were placed in 24-well cell culture trays containing some fresh wheat leaf.

To investigate the direct effects of PSP on the larvae midgut, the larvae were fasted for 8 h prior to the *in vitro* study.

Stock solutions of PSP and PSE were diluted with 32 K solution before use. The final DMSO concentration was less than 0.1% in the bath chamber.

### Midgut isolation

Midgut isolation was performed as described previously^31^. The larvae were pinned with the ventral side down on an ice bag, and the body wall was cut with fine microdissection scissors on the dorsal side along with the longitudinal axis from the last abdominal segment to the first thoracic segment. The midguts were transected at each end and rinsed with the 32 K solution. The peritrophic membrane with its food content and the Malpighian tubules were removed using forceps.

### Membrane potential measurements

Experiments were conducted as described previously by Peyronnet *et al*.^27^ with minor modifications. When the midgut was cut transversely, both ends tended to curl back onto themselves. The bathing medium was oxygenated by vigorous O_2_ bubbling for 30 min prior to use. To measure the apical membrane potential (*V*_a_), the isolated midgut was aspirated from its posterior end into a glass pipette until its anterior end curled around the pipette tip, thus exposing the apical surface (facing the lumen) of the epithelial cells. The pipette was lowered to near the bottom of the testing chamber, and the midgut epithelial cells were impaled with a glass microelectrode filled with 1 M KCl. To measure the basolateral membrane potential (*V*_bl_), each end of the midgut was aspirated into a holding pipette, and the epithelial cells were impaled from the basal side (facing the hemolymph). The electrode resistance was 50 to 150 MΩ. A disc electrode with lead wires (E242; Warner Instruments, Hamden, CT, USA) served as the ground electrode placed in the bath solution. The midgut membrane potentials were measured with an Axoclamp 900 A (Axon Instruments, Foster City, CA, USA) and a Digidata 1440 A interface (Axon Instruments); the pCLAMP 10.2 software (Axon Instruments) was used for data acquisition and analysis. In all experiments, the bathing solution was adjusted to maintain a potassium concentration equal to that of the 32 K solution. All experiments were performed at room temperature.

The midgut preparation was immersed in 3 mL of the 32 K bath solution *in vitro*. The membrane voltages were considered stable when *V*_a_ and *V*_bl_ did not change by more than 0.5 and 0.1 mV/min, respectively. After recording a stable control membrane voltage for 5 min, 0.4 mL of the 32 K solution was collected with a pipette, and an equal volume of the 32 K solution containing test compounds of interest was directly added to the bath. Measured voltages (*V*) were normalized relative to the voltage measured after the first 5 min (*V*_0_).

After different treatment periods (0, 2, 4, 6, 8, and 12 h), the force-fed larvae were dissected. The midgut was isolated and rinsed with 3 mL of the 32 K solution. When the membrane voltage remained stable for 5 min, we recorded the membrane potential *V*_a_ or *V*_bl_. The transepithelial voltage *V*_t_ is the difference between *V*_bl_ and *V*_a_. Electrophysiological data are presented as the means ± SEM for more than seven independent experiments.

Statistical analyses were conducted using two-tailed unpaired Student’s *t* test, and P < 0.05 was considered statistically significant.

## Additional Information

**How to cite this article**: Wang, Y. Y. *et al*. Effects of Periplocoside P from *Periploca sepium* on the Midgut Transmembrane Potential of *Mythimna separata* Larvae. *Sci. Rep.* **6**, 36982; doi: 10.1038/srep36982 (2016).

**Publisher’s note:** Springer Nature remains neutral with regard to jurisdictional claims in published maps and institutional affiliations.

## Figures and Tables

**Table 1:** Effects of PSP on the *V*_t_ of midgut epithelial cells from *M. separata* larvae.

Time/h	*V*_t_/mV		
	PSP	PSE	Cry1Ab
0	52.1	52.8	55.2
2	32.1	55.7	3.7
4	13.1	55.6	0
6	0.1	54.8	0
8	1.2	54.3	0

Note: *V*_t_(mV) = *V*_bl_−*V*_a_.
